# Impact of fasting on ^18^F-fluorocholine gastrointestinal uptake and detection of lymph node metastases in patients with prostate cancer

**DOI:** 10.1186/s13550-015-0159-2

**Published:** 2016-01-06

**Authors:** Maurits Wondergem, Friso M. van der Zant, Remco J. J. Knol, Jan Pruim, Igle J. de Jong

**Affiliations:** Department of Nuclear Medicine, Medical Center Alkmaar, Wilhelminalaan 12, 1815 JD Alkmaar, The Netherlands; Department of Urology, University Medical Center Groningen, University of Groningen, Groningen, The Netherlands; Department of Nuclear Medicine and Molecular Imaging, University Medical Center Groningen, University of Groningen, Groningen, The Netherlands; Department of Nuclear Medicine, Tygerberg Hospital, Stellenbosch University, Stellenbosch, South Africa

**Keywords:** ^18^F-fluorocholine, PET/CT, Prostate cancer, Urology, Patient preparations

## Abstract

**Background:**

^18^F-fluorocholine PET/CT is used to detect lymph node metastases in prostate cancer patients. Physiological ^18^F-fluorocholine in the gastrointestinal tract, especially in the intestines, may interfere with the detection of malignant lymph nodes. Fasting is frequently proposed in literature; however, scientific support is lacking. This study aims to determine the impact of fasting on ^18^F-fluorocholine uptake in the gastrointestinal tract.

**Methods:**

Eighty patients were studied, 40 fasted for at least 6 h prior to ^18^F-fluorocholine administration while the other 40 did not fast. ^18^F-fluorocholine uptake pattern and intensity were evaluated in the intestine near the abdominal aorta and four regions near the iliac arteries. ^18^F-fluorocholine intensity was also measured in the liver, pancreas, stomach and spleen.

**Findings:**

No statistically significant differences were found in ^18^F-fluorocholine uptake in the gastrointestinal tract between the fasting and non-fasting group.

**Conclusions:**

Fasting for 6 h has no effect on ^18^F-fluorocholine uptake in the gastrointestinal tract. Therefore, no effects on the detection of malignant lymph nodes are expected, and fasting is not recommended in our opinion.

## Introduction

Choline-PET/CT is widely used in patients with prostate cancer. Clinically, the imaging technique is used mainly for restaging of patients with a biochemical relapse and less frequently for initial staging. One of the strengths of choline-PET/CT is the detection and localisation of prostate cancer metastases in patients with slightly elevated PSA. Lymph node metastases can be detected in lymph nodes, which do not show malignant characteristics on conventional imaging such as CT and MRI. Patients with limited lymph node metastases may be selected for procedures with curative intent, while patients with more extensive disease can be protected from the morbidity of senseless aggressive therapies.

Detection of lymph node metastases may be hampered by high physiological choline uptake in the gastrointestinal tract commonly seen on choline-PET/CT. In the literature, fasting is frequently proposed before ^18^F-fluorocholine PET/CT, as can be found in a summary of literature by Chondrogiannis et al. [1]. Four hours of fasting before administration of ^11^C-choline is suggested in a document of the Society of Nuclear Medicine and Molecular Imaging [2]; however, both the Society of Nuclear Medicine and Molecular Imaging and the European Association of Nuclear Medicine have no specific guidelines for ^18^F- or ^11^C-choline PET/CT. To the best of our knowledge, there is no evidence that supports fasting prior to choline-PET/CT in order to suppress physiological choline uptake in the gastrointestinal tract. In a small animal study, fasting was not found to affect tracer uptake in the liver for both ^11^C-choline and ^18^F-fluorocholine [3]. This study aims to determine the impact of fasting, for at least 6 h, on ^18^F-fluorocholine uptake in the gastrointestinal tract.

## Methods

From May 2013 data of all ^18^F-fluorocholine-PET/CTs at our department were prospectively entered in a database. All patients gave written consent for the use of their anonymous data. Initially, all patients were scanned without prior fasting. Due to the observed high physiological ^18^F-fluorocholine uptake in the intestine, the patient preparation protocol for prostate cancer patients was changed after the first 40 patients had been scanned. In the revised patient preparation protocol, patients were asked to fast for at least 6 h prior to administration of ^18^F-fluorocholine. All scans were evaluated after a second cohort of 40 patients was scanned that had properly followed the revised patient preparation protocol.

LIST-mode data of the prostatic region were acquired for 10 min directly after ^18^F-fluorocholine administration (190 MBq, mean), followed by late images from the inguinal region to the base of the skull approximately 45 min later (Siemens Biograph-16 TruePoint PET/CT, Siemens Healthcare, Knoxville, USA).

For each patient, the LIST-mode data was used to determine the choline time-activity pattern in the intestine during the first 10 min after ^18^F-fluorocholine administration (software: Siemens syngo.via, Siemens Healthcare, Knoxville, USA). The Kolmogorov-Smirnov test was used to check for a normal distribution in the data. A *T* test or the Mann-Whitney *U* test was used to check for the differences between the groups at each time point after injection. A *T* test was used when the data in both groups showed a normal distribution; otherwise, the Mann-Witney *U* test was used.

Late data was used to determine the ^18^F-fluorocholine uptake in the liver, spleen, stomach, pancreas and intestine. In five different regions: 1 cm or less in the proximity of the aorta, both the left and right common iliac arteries, and both the left and right external iliac arteries, intestinal activity were measured. A *T* test or the Mann-Whitney *U* test was used to check for the differences between the groups in measured SUV_max_ and SUV_peak_, for each evaluated organ/region.

## Findings

### Patient population

In both the fasting and non-fasting group, 40 patients were included. Mean age was 71.9 years (range 59–83) and 69.1 (range 51–83) for the fasting and non-fasting group, respectively. Median PSA was 5.6 (0.3–194) and 4.3 (1.0–43.6), respectively. Indication for ^18^F-fluorocholine PET/CT was primary staging in six patients and three patients, biochemical relapse in 33 and 37 patients and other in one patient and zero patients for the fasting and non-fasting group, respectively.

### Early dynamic phase

**Fig. 1:**
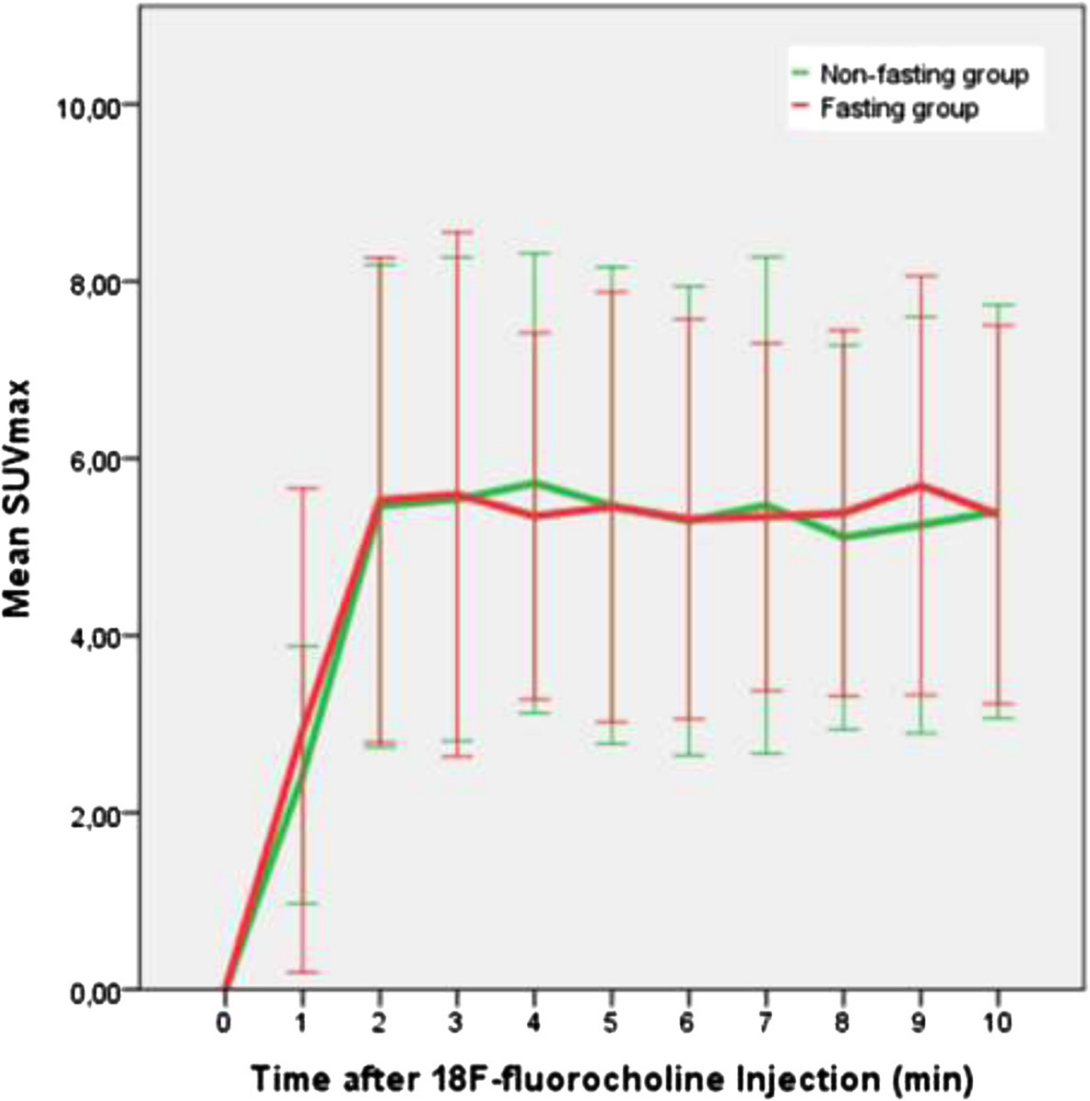
Mean intensity and uptake pattern of ^18^F-fluorocholine in intestine in the pelvis in the first 10 min after intravenous ^18^F-fluorocholine administration in both the fasting and non-fasting group. Error bars showing the standard deviation

^18^F-fluorocholine PET/CT shows a rapid uptake of the ^18^F-fluorocholine in the intestine in the first 2 min followed by a more or less stable concentration. No statistical differences were found between the fasting and non-fasting groups at any time-point (Fig. 1).

### Late phase

**Fig. 2:**
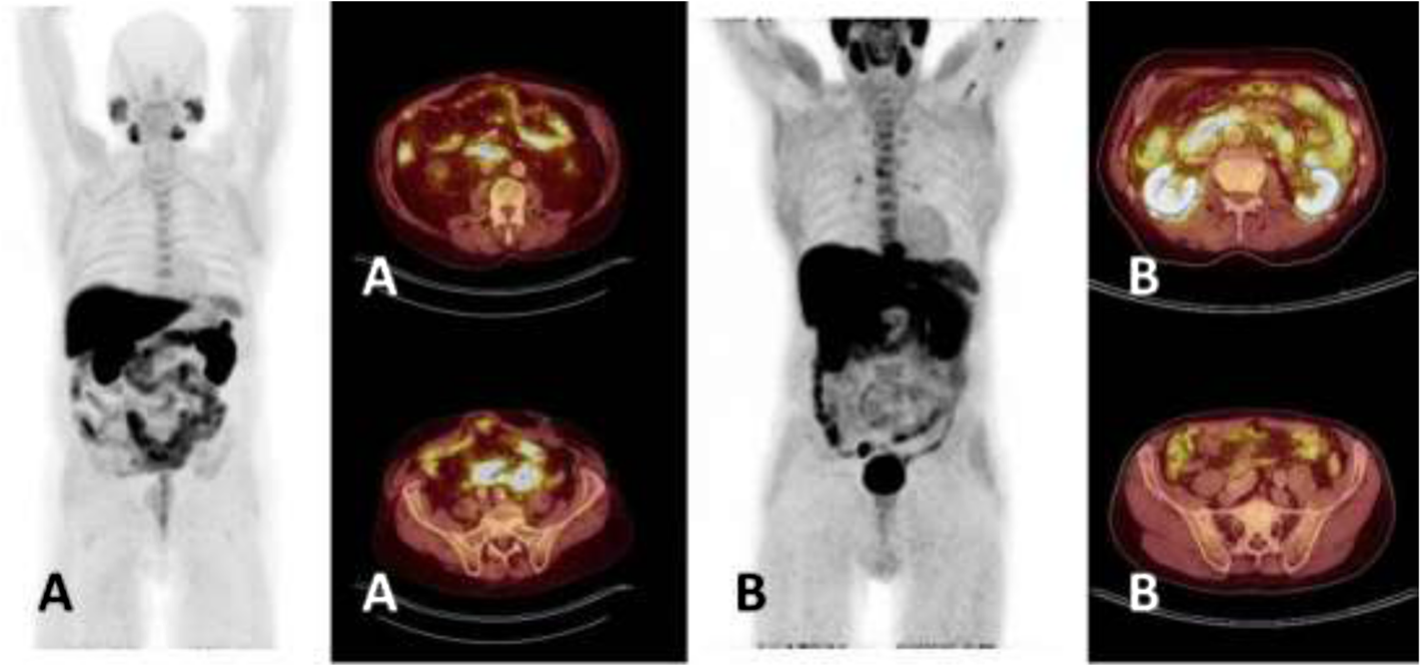
Example of ^18^F-fluorocholine PET/CT of a patient from the non-fasting group (**a**) and a patient from the fasting group (**b**). The fused images show transaxial views at the level of the abdominal aorta and the common iliac arteries

In all patients’ intestinal activity, which possibly interfered with lymph node metastases detection, was found near the aorta, especially in the duodenum. Possible interfering activity was found near the right common iliac artery in 65 and 80 % of the patients for the non-fasting and fasting group, respectively, right external iliac artery (93 and 98 %) and left external iliac artery (68 and 80 %). Activity near the left common iliac artery was found less frequently (37 and 45 %). The intensity of ^18^F-fluorocholine uptake in these regions was not significantly different between the fasting and non-fasting groups (Table 1, Fig. 2). In general, the intestinal activity near the aorta is higher than in the other regions, 0.003 ≥ *p* ≥ 0.000 for SUV_max_ and 0.002 ≥ *p* ≥ 0.000 for SUV_mean_ (ANOVA with post hoc Scheffe’s test). No significant differences were found between the activities in the other regions.

**Table 1:** Intensity of ^18^F-fluorocholine uptake in the intestine in five perivascular regions in pelvis and abdomen for fasting and non-fasting group

	Fasting	Number	Mean	*σ*	*p*
SUV_max_ intestine in region near					
Abdominal aorta	No	40	5.69	1.43	0.421*
	Yes	40	5.25	1.69	
Right common iliac artery	No	26	3.27	1.79	0.876^#^
	Yes	32	3.25	1.56	
Left common iliac artery	No	15	4.66	2.48	0.075*
	Yes	18	3.64	1.59	
Right external iliac artery	No	37	3.65	1.50	0.954^#^
	Yes	39	3.58	1.21	
Left external iliac artery	No	27	3.88	2.05	0.909^#^
	Yes	32	3.50	1.41	
SUV_peak_ intestine in region near					
Abdominal aorta	No	40	4.91	1.24	0.226*
	Yes	40	4.44	1.37	
Right common iliac artery	No	26	2.78	1.55	0.784^#^
	Yes	32	2.77	1.33	
Left common iliac artery	No	15	3.98	2.03	0.115*
	Yes	18	3.11	1.39	
Right external iliac artery	No	37	2.93	1.08	0.909^#^
	Yes	39	2.99	1.05	
Left external iliac artery	No	27	3.14	1.63	0.982^#^
	Yes	32	2.88	1.05	

Statistical analysis with *T test and ^#^Mann-Whitney *U* test

No significant differences were found in the ^18^F-fluorocholine activity uptake in the liver, spleen, stomach and pancreas between the fasting and the non-fasting groups (Table 2).

**Table 2:** Intensity of ^18^F-fluorocholine uptake in pancreas, liver, spleen and stomach for fasting and non-fasting group

	Fasting	Number	Mean	*σ*	*p*
SUV_max_					
Pancreas	No	40	9.39	2.10	0.661*
	Yes	40	9.14	2.83	
Liver	No	40	11.04	2.34	0.165*
	Yes	40	11.78	2.39	
Spleen	No	40	4.72	0.65	0.326*
	Yes	40	4.91	1.01	
Stomach	No	40	7.15	1.79	0.952*
	Yes	40	7.12	1.78	
SUV_peak_					
Pancreas	No	40	8.41	1.81	0.526*
	Yes	40	8.10	2.51	
Liver	No	40	10.21	2.27	0.128^#^
	Yes	40	10.87	2.19	
Spleen	No	40	4.25	0.54	0.620^#^
	Yes	40	4.45	0.95	
Stomach	No	40	6.34	1.74	0.860*
	Yes	40	6.27	1.62	

Statistical analysis with *T test and ^#^Mann-Whitney *U* test

## Discussion

**Fig. 3:**
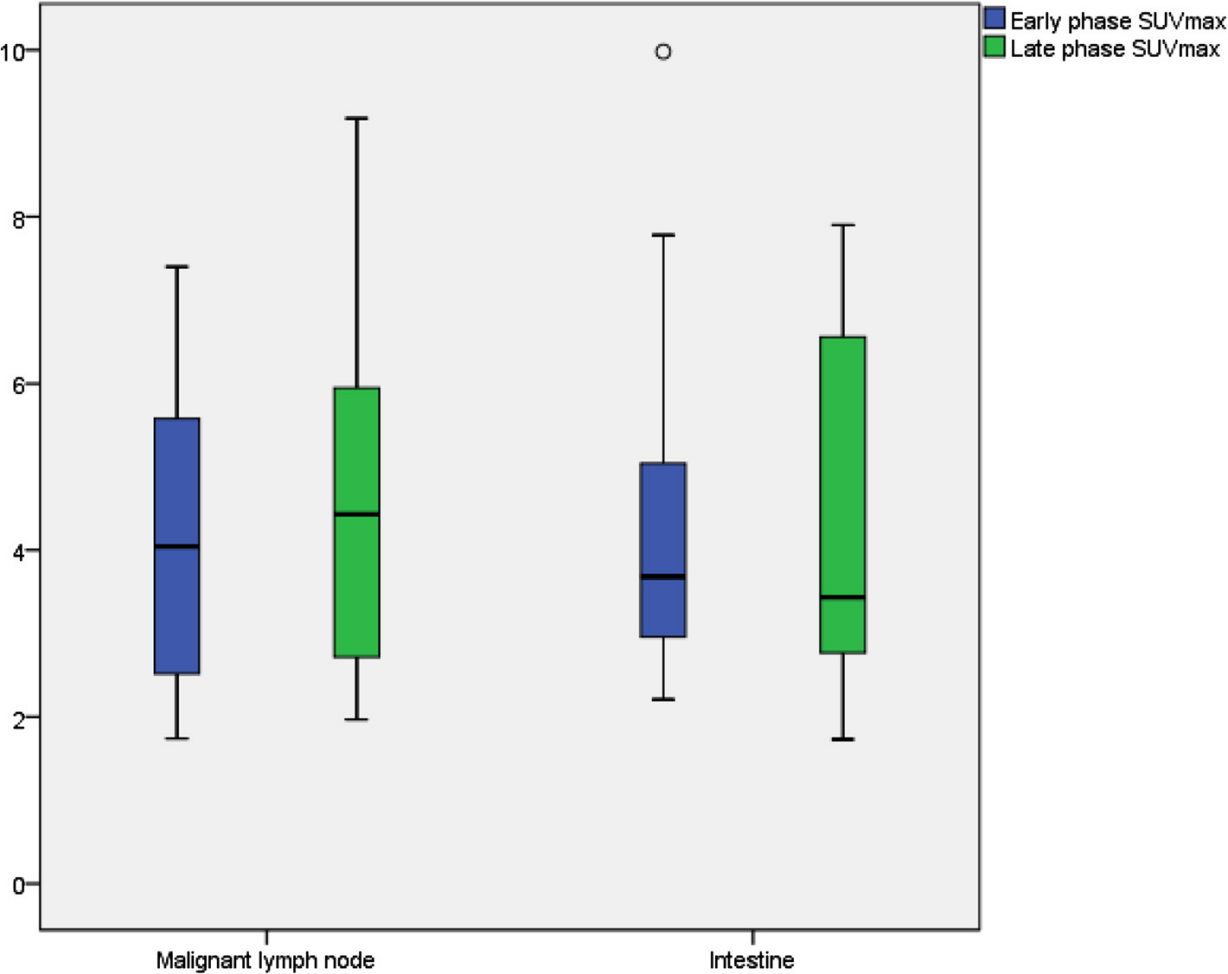
Boxplots of intensity (SUV_max_) of ^18^F-fluorocholine uptake in malignant lymph nodes and intestine in the early and late phase. No statistically significant difference was found. Early phase *p* = 0.823 and late phase *p* = 0.897 (Mann-Whitney *U* test). Boxplots showing the median, first and third quartile, maximum and minimal values not considered an outlier and outliers (>1.5 interquartile range)

The data of our study shows that physiological intestine activity in the vicinity of the abdominal aorta and iliac arteries, regions in which lymph node metastases of prostate cancer are frequently found, is present in all prostate cancer patients receiving ^18^F-fluorocholine PET/CT, especially near the abdominal aorta. In earlier data from our group, we found in 100 patients that the activity (SUV_max_) in malignant lymph nodes and physiological intestine activity does not significantly differ at both early and late data acquisition time points (Fig. 3, unpublished). Therefore, interference of physiological activity in the intestine with detection of lymph node metastases is realistic.

Most papers on acquisition protocols of ^18^F-fluorocholine deal with the time-point of acquisition and with the effects of voiding and hydration on detection of lesions in the prostate. Fasting is regularly proposed in acquisition protocols without scientific support [1]. Our data show that the nutritional status has no impact on the uptake of ^18^F-fluorocholine in the gastrointestinal tract and, more specifically, in regions in the gastrointestinal tract that may interfere with the detection of lymph node metastases.

The CT part of the PET/CT is important to detect lymph nodes in areas with high intestinal activity. To distinguish lymph nodes in these areas, acquisition of CT images of diagnostic quality with administration of intravenous iodinated contrast is necessary. Once lymph nodes are detected on CT, the combination of PET and CT may distinguish malignant lymph nodes from physiological lymph nodes, however, especially in small lymph nodes, characterisation may remain difficult.

The statistical power may be a shortcoming of the study. In all regions, the observed differences in the mean SUV_max_ and SUV_peak_ are relatively small in comparison to the observed standard deviation. One could suggest that the large study populations would be needed to exclude the presence of statistically significant differences. However, in our opinion, the clinical value of such findings would still be irrelevant. Regarding the overlap of found ^18^F-fluorocholine intensity in all intestine regions in both groups with the intensity in malignant lymph nodes, no effects on the detection of lymph node metastases are expected.

## Conclusion

Intestinal activity that possibly interferes with the detection of lymph node metastases is encountered in all prostate cancer patients receiving ^18^F-fluorocholine PET/CT. Fasting for at least 6 h before administration of ^18^F-fluorocholine does not influence the uptake of ^18^F-fluorocholine in the gastrointestinal tract in these patients. Therefore, fasting is not recommended in our opinion.

### Ethical approval

All procedures performed in studies involving human participants were in accordance with the ethical standards of the institutional and/or national research committee and with the 1964 Helsinki declaration and its later amendments or comparable ethical standards.

### Informed consent

Informed consent was obtained from all individual participants included in the study.

## References

[1] Chondrogiannis S, Marzola MC, Grassetto G et al. New acquisition protocol of ^18^F-choline PET/CT in prostate cancer patients: review of the literature about methodology and proposal of standardization. BioMed Res Int. 2014; doi10.1155/2014/215650. 10.1155/2014/215650 PMC4119889 25121090

[2] ^11^C-Chlonine. SNMMI PET Center of Excellence and the Center for Molecular Imaging Innovation & Translation. http://snmmi.files.cms-plus.com/Choline%20chloride%20C-11%20(3).pdf.

[3] Kolthammer JA, Corn DJ, Tenley N (2011). PET imaging of hepatocellular carcinoma with ^18^F-fluoroethylcholine and ^11^C-choline. Eur J Nucl Med Mol Imaging..

